# Intimate partner violence and preschool self-regulation: Examining the role of maternal emotion socialization in Black families

**DOI:** 10.1111/sode.12691

**Published:** 2023-06-15

**Authors:** Renee Lamoreau, Jae eun Park, Hilary Skov, Allison Pequet, Sarah A. O. Gray

**Affiliations:** 1 Department of Psychology, Tulane University School of Science and Engineering, New Orleans, USA; 2 Department of Psychology, University of Notre Dame College of Arts and Letters, Notre Dame, USA

**Keywords:** Black/African American, early childhood, emotion socialization, intimate partner violence, parenting, self-regulation

## Abstract

The ways that parents respond to children’s negative emotions shape the development of self-regulation across early childhood. The objective of this study was to examine child self-regulation in the context of intimate partner violence (IPV) exposure in a sample of Black, economically marginalized mothers and their young children (aged 3–5 years, *N* = 99). The study investigates the conditional effects of emotion socialization practices that (1) encourage expression of and problem-solving around negative affect (“supportive”), and (2) encourage suppression of affective displays (“suppressive”) on children’s self-regulation. We found a significant association between higher child self-regulation and supportive parental reactions in the context of psychological IPV. We also found a significant association between higher child self-regulation and suppressive parental reactions in the context of psychological IPV. Our findings are consistent with prior research suggesting Black parents who teach varied strategies for emotional expression may promote children’s adaptation in high-stress family environments. Macrosystem factors such as systemic racism and discrimination as well as the threat of family violence may shape how parents approach emotion socialization and the teaching of affective self-expression and self-regulation.

## 1 | INTRODUCTION

Interactions with caregivers serve as the primary modality through which young children learn about emotions (Denham et al., 2015; Friedlmeier et al., 2011; Zeidner et al., 2003). Caregivers do this in how they react to children’s emotional expressions, discuss emotions with their children, and express their own emotions. This process is called emotion socialization, a term that refers to how caregivers teach children to understand and regulate their emotions (Dunsmore & Halberstadt, 1997; Eisenberg et al., 1998).

The unique demands of ecological context, or the “macrosystem” in Bronfenbrenner’s (1977) model, is a critically important but often overlooked factor in shaping how parents approach emotion socialization. Cultural norms within the US influence how parents socialize their children’s emotional experience (Brown et al., 2015; Cole & Tan, 2007; Friedlmeier et al., 2011). In contexts of racial marginalization and violence, parents must grapple with the reality that some negative emotional expressions are dangerous. Given the pressures of historical racial discrimination, Black families are more likely than White families to teach children emotional suppression strategies that protect them in racially biased situations outside of the home (Dunbar et al., 2017; Dunbar, Zeytinoglu, et al., 2022; Nelson et al., 2012; Thomas & Blackmon, 2015).

When families are simultaneously experiencing the threat of violence *within* the home in the form of intimate partner violence (IPV), this also puts unique demands on emotion socialization. Such an environment is evocative of negative emotions and taxes children’s emerging self-regulation skills (Bender et al., 2022; Samuelson et al., 2012). Children exposed to IPV may demonstrate better outcomes when they are given the skills to manage strong emotions (Katz et al., 2020), but family violence may also be associated with less parental toleration for the expression for negative affect (Katz et al., 2007). Children exposed to IPV often display challenges with emotional dysregulation that place them at risk for later behavioral and psychological problems (Harding et al., 2013). Ultimately, the threat of violence both *inside* and *outside* of the home is an important consideration when examining the impacts of emotions socialization on children’s regulatory outcomes.

### 1.1 | Emotion socialization and self-regulation

Parents’ emotion socialization of children serves an adaptive and functional role by helping young children learn to self-regulate (Zeman et al., 2013), defined as a process involving the monitoring and control of thoughts, behaviors, emotions, and actions (Carlson, 2003). Parent–child interactions serve as an important context through which children learn how to modulate their emotional expression, thinking, and behavior (Grusec, 2002). A significant body of literature has established a link between parenting behaviors and children’s self-regulatory capacities (Eisenberg et al., 2010; McCabe et al., 2004). Specific parenting behaviors that facilitate the development of adaptive self-regulation include parental scaffolding, cognitive stimulation at home, sensitivity, autonomy support, and effective disciplinary practices (Bernier et al., 2010; Fay-Stammbach et al., 2014; Whipple et al., 2011). By responding to children’s emotional expressions, parents help children to manage themselves in socially appropriate and context-specific ways (Morris et al., 2007).

The ability to regulate one’s emotions and behavioral responses is generally associated with good mental health and well-being (Gross & Muñoz, 1995), whereas dysregulation is associated with psychopathology (Eisenberg et al., 1998). Early childhood represents a period of rapid growth in self-regulatory abilities, underscoring the importance of early parent-child interactions during this window of development (Bernier et al., 2010; Fonagy & Target, 2002), especially in contexts of stress (Blair, 2010). Early self-regulatory abilities contribute to school readiness by helping children sustain attention in learning activities and adjust to new expectations (Blair&Raver, 2015), developmental competencies that come online during the preschool age range (Montroy et al., 2016). When children enter formal schooling for the first time, early self-regulation is linked with literacy, math, vocabulary, and adaptive classroom behaviors (Mcclelland et al., 2007; Rimm-Kaufman et al., 2009). Early self-regulation is also linked with long-term outcomes in adolescence and adulthood. Mofitt et al. (2011) found that childhood self-control predicted physical health, substance abuse, financial security, and criminal activity in a large-scale longitudinal study following children over a 30-year period.

### 1.2 | A functionalist approach to emotion socialization in Black families

Parents have varied goals when teaching children about the expression and restriction of emotion. Although some parents believe that emotions should be controlled and not expressed, other parents may believe that emotions should be expressed and support their children in expressing them in socially acceptable ways (Gottman et al., 1996). The difference is particularly relevant when thinking about how parents respond to children’s expression of negative emotion. Eisenberg et al. (1998) distinguishes between parental responses to children’s negative emotional expressions that are “supportive” versus “unsupportive.” Unsupportive responses typically involve punitive responses, minimizing responses, and/or expressing parental distress. In predominantly White samples, unsupportive parental responses have been associated with heightened emotional arousal and greater risk of poor emotional coping and depressive symptoms in their children (Sanders et al., 2015; Shaffer et al., 2012). Supportive parental responses involve emotionfocused responses, problem-focused responses, and expressive encouragement. Supportive parental responses may decrease children’s risk of emotion dysregulation and internalizing and externalizing problems (Eisenberg et al., 1998; Katz & Hunter, 2007). Overall, the emotion socialization literature has largely studied parental responses as serving binary functions, considering unsupportive responses to confer risk and supportive responses to promote positive outcomes. Though there is some heterogeneity here, as some researchers have examined response types separately (Halberstadt et al., 2008; Nelson et al., 2013)

A functionalist approach to emotion socialization recognizes how parents teach, model, and reinforce emotional expressions that meet the demands of the social and physical environment (Campos et al., 1989). From this perspective, all inferences about the functions of emotion can only be interpreted considering the unique demands of a particular environment. Functionalists assume that emotions are adaptations that ultimately promote social and physical survival (Keltner & Gross, 1999). Emotions serve both an intrapsychic and interpersonal function by helping individuals appraise threat in their environment and gain access to others’ emotional states (Bretherton et al., 1986). As children are repeatedly exposed to IPV, parents may engage in functionally adaptive socialization strategies to ensure children’s immediate safety.

A functionalist perspective is especially useful when considering how Black families living in poverty approach emotion socialization, and the context-specific ways that self-regulation may be protective for Black children. Racism, discrimination, and segregation have inevitably shaped the lives of Black children and families (Coll et al., 1996) and subsequently the ways that parents respond to children’s emotional expressions (Labella, 2018). Although race and economic status are distinct factors, because of historical and ongoing structural oppression (e.g., the legacy of slavery and Jim Crow, redlining, the school-to-prison pipeline), Black families are more likely to experience poverty when compared with White families (Beech et al., 2021; Burton, 2018; Darity Jr et al., 2022). There is evidence that the stressors of parenting in contexts of poverty are associated with parental emotion socialization strategies of minimization, punishment, and distress, with implications for children’s capacity to self-regulate (Shaffer et al., 2012). Cultural factors in Black families like the importance of maintaining social networks and group harmony as well as the interplay between spirituality and emotional expression are also important considerations (Harrison et al., 1990; Parker et al., 2012). Therefore, parental emotion socialization strategies, and their effects on children’s emotional and behavioral development, should be examined in specific cultural and ecological contexts.

Black families use a combination of supportive and suppressive socialization strategies in adaptive ways. In a systematic review of parental emotion socialization in Black families, Labella (2018) found that Black families simultaneously celebrate the expression *and* restriction of emotion. Black families may use socialization strategies that facilitate emotional and behavioral control, but also stress the importance of emotional expression, connectedness, and oral communication, which are rooted in traditional Afro-cultural values (Garrett-Peters et al., 2008, 2011). Further, Labella (2018) also reports that Black parents are more likely to react to their children in a way that would typically be categorized as “unsupportive” among White parents. Black parents are tasked with preparing children, particularly Black boys, to enter a mainstream society that perceives negative emotional expressions as threatening (Thomas & Blackmon, 2015), a setting that Lozada et al. (2022) deems an emotionally inhibiting environment. “Unsupportive” practices that downplay or punish expressions of anger, for example, may therefore be adaptive in the face of racial discrimination. This aligns with other literature examining a “no-nonsense” style of parenting combining high levels of warmth and coercive control that more broadly characterize many Black families (Brody & Flor, 1998). In sum, these findings affirm that Black parents use emotion socialization in context-specific and culturally relevant ways that encourage expression but also prepare children for bias (Dunbar, Lozada, et al., 2022; Dunbar, Zeytinoglu, et al., 2022).

Dunbar et al. (2017) propose a model which integrates emotion and racial socialization and challenges some of the traditional notions of supportive versus unsupportive emotion socialization practices. According to this model, Black families may promote context-specific suppression strategies (e.g., telling children to control their anger) to protect their children during interracial interactions and bias incidents. Dunbar et al. (2017) labeled such an adaptation “emotion-centered racial coping,” which includes suppression response to children’s negative emotions and direct discussion of race-related conflicts. Similar to what Labella (2018) concluded, emotion socialization practices like suppression or minimization that have been labeled as “unsupportive” may actually be protective for Black children. Since expression of negative affect leads to higher rates of discrimination, Black mothers may be less supportive and more controlling to their children’s negative emotional displays to prepare children for racism and bias during interracial interactions (Dunbar, Zeytinoglu, et al., 2022; Lozada et al., 2022; Nelson et al., 2012).

### 1.3 | Family conflict

Another important context for emotion socialization is family conflict and IPV, which includes a range of abusive behaviors such as physical aggression, psychological, control and manipulation, and coercive sexual behaviors (Breiding et al., 2015). Black women are disproportionately exposed to IPV (Taft et al., 2009) and are more likely to be murdered by an intimate partner (Petrosky et al., 2017) when compared with women from other racial and ethnic groups. Based on data from the 2010 National Intimate Partner and Sexual Violence Survey (NISVS), at least one third (35%) of non-Hispanic White women have been a victim of rape, physical violence, or stalking by an intimate partner in their lifetime (Breiding et al., 2014). Significantly more (44%) non-Hispanic Black women in the U.S. reported the same lifetime IPV exposures. Furthermore, poverty is a risk factor for IPV (Butchart et al., 2010), and Black women who simultaneously experience poverty and IPV are an especially vulnerable group (Gillum, 2019).

IPV is inherently a fear-inducing and dysregulating experience for young children. When children witness an attachment figure being victimized, their internal sense of safety is disrupted (Levendosky et al., 2012). This experience is uniquely traumatizing and emotionally activating for young children. IPV also alters how parents interact with their children and how they respond to their emotional expressions. One mechanism through which this may occur is via a “spillover” of interparental conflict into parenting processes, leading to interactions that lack sensitivity or responsiveness (Erel & Burman, 1995). This “spillover” has enduring consequences for children’s social, cognitive, emotional and behavioral functioning (Chiesa et al., 2018). Children who have witnessed IPV are at increased risk for internalizing problems such as depression and anxiety (Kernic et al., 2003), and externalizing problems such as aggressive behavior and conduct problems (Evans et al., 2008; Fantuzzo et al., 1991).

The process by which IPV exposure leads to negative outcomes for children may be related to regulatory deficits. Exposure to IPV may induce elevated physiological arousal and negative affect, and chronic exposure may expend the child’s affective and physiological resources, overwhelming their regulatory capacities over time (Davies & Cummings, 1998). Although IPV exposure has been linked with deficits in emotion regulation (Bender et al., 2022; Carpenter & Stacks, 2009), less is known about the effects of IPV on more integrated or global measures of self-regulation, especially during early childhood. Given that emotion regulation is a subdomain of self-regulation, IPV may produce parallel deleterious effects on broader measures of self-regulation. A recent review by Bogat et al. (2023) proposes that child self-regulation is a key mediator linking IPV with children’s negative adjustment outcomes. IPV may interfere with the normative process of coregulation of distress between parents and children, leading to disruptions in the development of self-regulation during sensitive periods of brain development. Self-regulation is increasingly recognized as a transdiagnostic factor underlying child mental health, academic functioning, and social functioning (Bogat et al., 2023).

Of any age group, children under the age of 6 years old are also at the greatest risk of direct IPV exposure (Fantuzzo & Fusco, 2007). Early childhood is a sensitive period in which caregiving experiences shape the development of foundational cognitive and social-emotional competencies like self-regulation (McClelland et al., 2018; Montroy et al., 2016). Violence exposure occurring during early childhood, when neuronal connections are rapidly proliferating and then being “pruned” in response to environmental experiences, may be particularly harmful to long-term outcomes (McCoy, 2013; Shonkoff et al., 2000).

### 1.4 | Emotion socialization as a moderating trauma process

Emotion socialization may function as a buffer against the adverse effects of violence exposure on children. Drawing from a relational PTSD framework, the primary caregiving relationship serves an important function in managing threat and stress in young children (Scheeringa & Zeanah, 2001). Caregiving behaviors may have a moderating effect by either intensifying or reducing the effect of violence on children. Parenting characterized by warmth, responsiveness, and appropriate discipline can be a source of resilience for children who have witnessed IPV (Graham-Bermann et al., 2009). Positive parent-child interactions may strengthen the security of attachment relationships, which also facilitate the development of adaptive emotion regulation and prosocial skills in the context of IPV (Howell et al., 2010). In some cases, mothers may even attempt to overcompensate for family violence by being more attentive and responsive with their children, which has a buffering effect on child outcomes (Levendosky et al., 2003).

Some parents experiencing IPV may struggle with talking to children about emotions like anger or fear (Katz & Windecker-Nelson, 2006), possibly because they themselves are emotionally dysregulated or have difficultly modulating strong, negative emotions. Other parents can separate their experiences of IPV from their interactions with their children and tolerate a wider range of emotional expression in their children. In general, IPV-exposed children fare better when their parents support them in talking about and expressing feelings (Fogarty et al., 2021; Howell, 2011; Katz et al., 2020). For example, Katz and Windecker-Nelson (2006) found that emotion coaching moderated the relationship between IPV and child behavior problems. When mothers provided high levels of emotion coaching, there was no relationship between IPV and children’s behavior problems, suggesting that emotion coaching buffered the adverse effects of IPV by supporting children’s processing of negative emotions. It is important to note that the sample was predominately White (86%), middle class families. Replicating this work with Black mothers and children is critical, not only because they are underrepresented in this literature, but also because, in addition to increased risk of IPV exposure, they are at risk of experiencing the stressors of poverty and racism simultaneously (Mushonga et al., 2021).

Thus, the present study examines the co-contribution of exposure to IPV and parental emotion socialization in a sample of Black-identified, low-income and economically marginalized mothers and young children.

### 1.5 | Self-regulation and coping in contexts of violence

In violent and unsafe familial contexts, where the goal is safety and survival, children develop adaptive coping and regulatory processes. Functional adaptation posits that individuals develop specific skills and abilities to adapt and survive in their proximal environments (Wadsworth, 2015). Research with children experiencing IPV indicates that avoidant and suppressive coping strategies can be adaptive in contexts of high stress. For example, O’Brien et al. (1995) found that IPV-exposed children had better mental health outcomes when they used avoidant and withdrawing coping strategies (e.g., physically removing themselves from the conflict, distracting with another activity). Similarly, Davies et al. (2015) found that children who were highly involved and emotionally reactive to parental conflict demonstrated more psychological maladjustment. Taken together, even though the literature indicates that children exposed to IPV fare better when they are given the opportunity to *express* negative emotions, it may be equally important for parents to also teach children to *suppress* and avoid negative emotions and/or interactions as an adaptive coping strategy.

### 1.6 | The present study

The present study examined whether and how maternal emotion socialization practices serve a protective function in the context of IPV. Specifically, this paper examines whether supportive or suppressive maternal emotion reactions moderate the association between children’s exposure to IPV and their self-regulation capabilities in a sample of Black mothers and preschool-aged children. It was hypothesized that supportive maternal emotion socialization practices would serve as a protective factor for self-regulation in the context of IPV. We tentatively hypothesized that suppressive parental emotion socialization strategies would serve as a risk factor for children’s self-regulation capacities, but based on the work of Dunbar et al. (2017) and Labella (2018), we considered that the opposite may be true, given that suppressive responses may also function as a protective factor for Black families.

## 2 | METHODS

### 2.1 | Participants

Data were collected from 158 mothers and their preschool-aged children recruited from local pediatric service agencies serving low-income families in the greater New Orleans area. All participating families were at or near the federal poverty level (≤185% federal poverty guidelines). For this analysis focused on IPV in Black mother-child dyads, 14 mothers were excluded for not having a romantic partner at data collection, 25 were excluded for not self-identifying as Black, and 20 mothers were missing one or more measures used in the analysis. The final analytic sample for this paper therefore consisted of 99 dyads, all of whom self-identified as Black or African American and were in romantic partnerships. Children were between the ages 3–5 years (*M* = 4.30, *SD* = .77), and the sample was evenly split between sex assigned at birth (51% female, 49% male). Mothers were 20–46 years old (*M* = 30.26, *SD* = 5.33), and reported varying levels of educational attainment (i.e., 35% had 1–3 years of college, 29% had a high school diploma or equivalent degree). Families were excluded from participation if the mothers were unable to complete study measures in English; if children had a previous diagnosis of global developmental delay per parental report; or if caregivers were not biological mothers, due to heritability of other outcomes of interest in the parent study (see Glackin et al., 2021).No Black-identified participants were excluded for their limited English proficiency.

### 2.2 | Procedures

Caregivers and their children were recruited from local Head Start preschools; Women, Infants, and Children Clinics; and through referrals from other study participants. Interested mothers completed a brief screening survey for sociodemographic information, as well as a checklist screener of exposure to potentially traumatic events (PTEs) for both themselves and their children (Life Events Checklist; Gray et al., 2004). Mothers were compensated $5 upon completion of screening surveys regardless of their participation in later study activities. To examine the impact of violence exposure across generations, families were oversampled for mother and child interpersonal violence exposure, such that all eligible mothers who endorsed themselves or their child witnessing or experiencing a violent PTE on the LEC (e.g., “physical attack or threats,” “being mugged or robbed”) were invited to participate in the study. Additionally, to ensure a range of exposures and experiences, a subset of mothers who did endorse exposure to violence in themselves or their child were also invited to participate.

Mother-child dyads were scheduled to complete a 2-hour visit which took place either in the families’ homes or on-site in our laboratory space, depending on the parent’s preference. Identical study procedures were used in both settings and there was no difference in the assessments used in this analysis by setting type. Informed consent was acquired and mothers reported on both their own as well as their child’s exposure to violence and other PTEs during structured interviews. Mothers were compensated with a $50 gift card, and the children received a small toy and/or book. All procedures were approved by the Tulane University Institutional Review Board. Data were collected by research lab members during both visits. Visits were facilitated by five graduate research assistants and two lab managers. Of the five graduate research assistants, three identified as White women, one identified as a Black woman, and one identified as a Latina woman.

## 3 | MEASURES

### Sociodemographic information.

Mothers completed a recruitment screener that included their own and their child’s age, race, ethnicity, education level, English proficiency, and marital status.

### Maternal IPV victimization.

Mothers reported on their exposure to IPV with partners in the home using the revised Conflict Tactics Scale (CTS-2; Straus et al., 1996). The CTS-2 is a comprehensive, self-report instrument used to measure the frequency and severity of IPV. The CTS-2 is adapted from the original Conflict Tactics Scale (CTS), which has been validated for use in diverse populations and cultures. The CTS-2 has been found to be a reliable and valid measure for use in Black and Hispanic Americans (Chapman & Gillespie, 2019). This study includes findings from two of the five CTS-2 subscales, psychological aggression and physical assault; sexual assault and negotiation were removed due to our interest in types of violence that children may be more likely to witness, and injury was removed due to concerns about mandated reporting. The CTS-2 requests respondents to report an approximate number of times an event occurred in the past twelve months. For the purpose of this analysis, only the victimization items were used for psychometric analyses. The items are rated on an 8-point frequency scale, ranging from never to more than 20 times. Mothers were also given the option to indicate the ever prevalence (whether the behavior has ever occurred) and annual incidence of IPV, as well as the chronicity and severity of the event. The items are summed using the midpoints of the response options to create subscale frequency totals, as per the authors’ guideline (Straus et al., 1996).

### Emotion socialization.

The Coping with Children’s Negative Emotions Scale (CCNES; Fabes et al., 2002) is a self-report questionnaire used to assess parental response to children’s negative emotions. The scale consists of 12 hypothetical scenarios in which their child is expressing negative emotions like sadness, fear, anger, disappointment, and embarrassment. Each of the 12 items may be responded in six theoretically distinct ways. Parents are asked to rate their response on a 7-point Likert scale (1 = very likely, 7 = very unlikely). The responses yield six subscales, which can be averaged to form composite measures of reactions to children’s expression of negative emotion and distress (DeBoard-Lucas et al., 2010; Nelson et al., 2009; Shaffer et al., 2012). The supportive reactions scale averaged scores from the expressive encouragement, problem-focused responses, and emotion-focused reactions subscales (*M* = 5.79, *SD* = 1.03, *α* = .93). The suppressive reactions scale averaged scores from the minimizing and punitive reactions subscales (*M* = 2.51, *SD* = .95, *α* = .89). Unlike in previous work that has used these two composite variables (e.g., Shaffer et al., 2012), we omitted the distress reactions subscale entirely and used the language “suppressive” rather than “unsupportive” to align with the Dunbar et al. (2017) conceptual model for emotion socialization in African American families. Furthermore, the distress reactions subscale has been omitted in other analyses because of it being a more “passive” emotion socialization construct (Mirabile, 2014) and because it may be more indicative of parents’ *own* emotion regulation (Rodas et al., 2016). Dunbar’s conceptual model considers both minimizing and punitive reactions to function as adaptive suppressive responses in African American families.

### Child self-regulation.

The Preschool Self-Regulation Assessment (PSRA; Smith-Donald et al., 2007) was used to measure children’s self-regulatory outcomes across a series of 10 structured tasks and an accompanying report of children’s (a) Positive Emotions and (b) Attention/Impulse Control during the assessor-child interaction. The assessor report has been validated for use in diverse socioeconomic and racial/ethnic populations (Daneri et al., 2018). Items on the assessor report were coded using a 4-point Likert scale ranging from 0 to 3 in response to 28 behavioral descriptors by two coders (30% double-coded; item ICCs = .63–.86). Both scales demonstrated very good internal consistency (Attention/Impulse Control, *α* = .96; Positive Emotion, *α* = .86) when analyzed using the methods cited by Smith-Donald et al. (2007). The present study used the Attention/Impulse Control subscale because of its clinical relevance and ecological validity; therefore, items measuring attention and impulse control were combined into a single behavioral self-regulation scale (*M* = 35.04, *SD* = 12.55, *α* = .96). In general, children with higher self-regulation ratings had better attentional skills and were better able to understand and follow directions. Children with lower ratings had more difficulty modulating their emotional responses, indiscriminately touching testing materials, and inhibiting impulses.

### Covariates.

Four covariates were identified a priori based on a review of the literature: children’s age, sex, maternal education, and maternal depressive symptoms. Children’s ages were recorded in months and were calculated using parent-reported birth date and the date of the data collection visit. Sex was coded 0 for male and 1 for female per parental report. Maternal education was reported on an ordinal scale ranging from “8th grade or less” (0) to “Professional degree (e.g., MD, JD, PhD)” (8). Maternal depressive symptoms were reported by mothers using a sum score from the Center for Epidemiologic Studies Depression Scale—Revised (CES-D-R; Van Dam & Earleywine, 2011), a 20-item scale assessing depressed mood, disturbances with appetite or sleep, difficulty concentrating, fatigue, feelings of worthlessness, psychomotor agitation, and suicidal ideation using a 4-point Likert scale. Sum scores were created for depressive symptoms which fell in the clinically significant range for this sample (*M* = 9.92; *SD* = 10.84).

## 4 | RESULTS

### 4.1 | Preliminary analyses and descriptive statistics

Data were examined for missing items and normality assumptions. We use bootstrapping in the main analyses to adjust for non-normality. Therefore, we did not use multiple imputation (MI) to impute missing data because bootstrapping and MI are non-compatible. A Missing Values Analysis indicated that Little’s (1988) test of Missing Completely at Random (MCAR) was not significant (*x*^*2*^ = 8.62, *p* = .97). Preliminary analyses examined descriptive statistics and correlations among study and demographic variables (see Table 1). Approximately one third (32%) of included mothers reported being victimized in at least one act of physical aggression, and the majority (82%) reported being exposed to at least one act of psychological aggression. Although physical assault and psychological aggression were conceptualized in separate models (described below), both models controlled for the other construct.

### 4.2 | Main analyses

**FIGURE 1:**
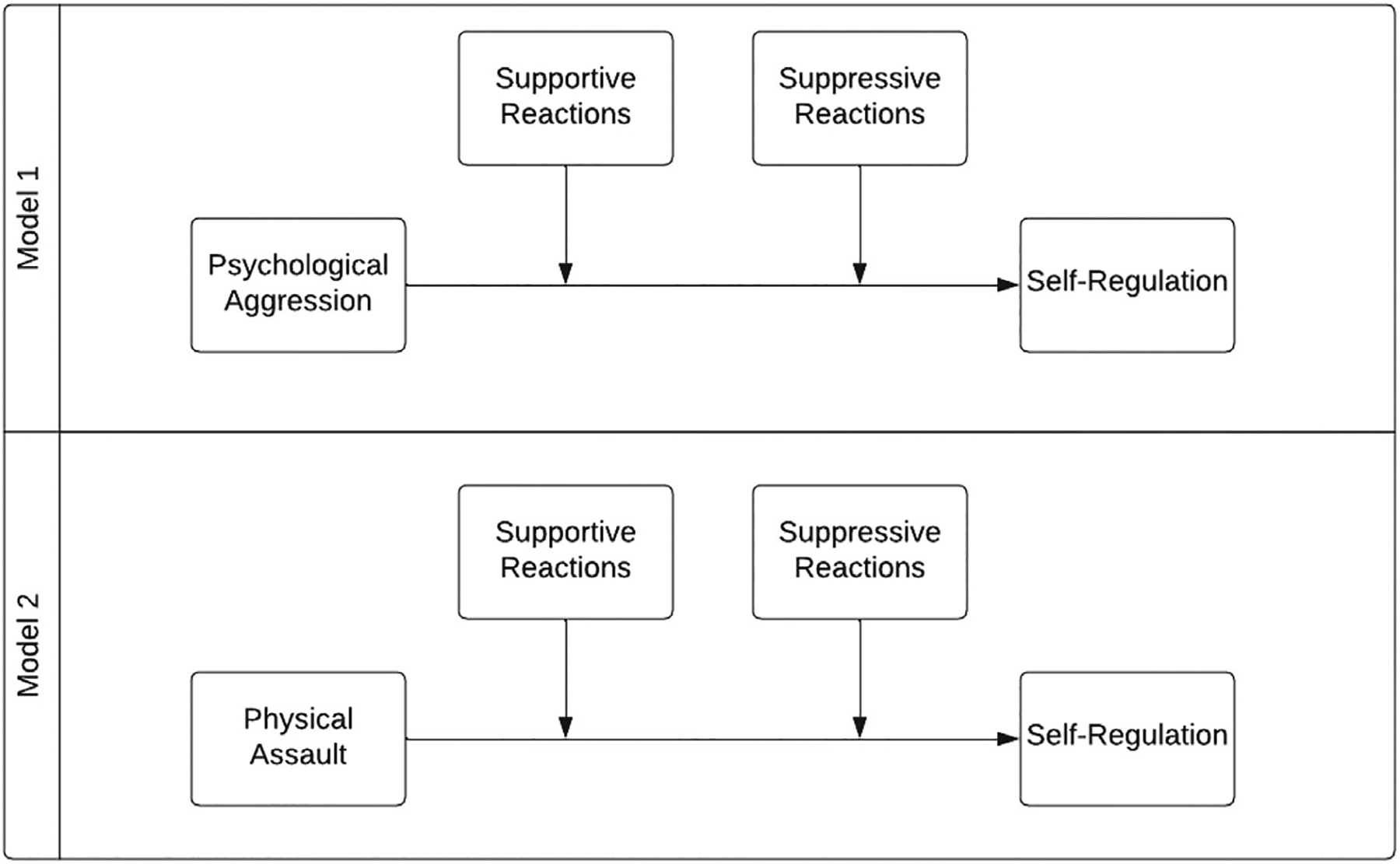
Conceptual model for double moderation analyses testing the co-contribution of supportive and suppressive reactions in the context of IPV.

We ran two bootstrapped additive multiple moderation regression models to examine the co-contribution of supportive and suppressive emotion socialization with both forms of IPV (physical assault and psychological aggression), with children’s observed self-regulation as the outcome variable. A particularly useful application of additive moderation, also known as “double moderation,” is when there is a single conceptual moderator (i.e., parental emotion socialization behaviors), but it is multicategorical (i.e., supportive responses versus suppressive responses) (Montoya, 2018). Unlike in 3-way interactions, the moderators in additive multiple moderation models do not interact with each other, though the effect of the independent variable on the dependent variable is conditional on both moderator variables (Hayes, 2017). Model 1 examined supportive and suppressive reactions in the context of psychological aggression; Model 2 examined supportive and suppressive reactions in the context of physical assault (see Figure 1 for conceptual diagram).

The PROCESS macro (Hayes, 2013), an observed variable OLS and logistic regression path analysis modeling tool that uses a bootstrapping method of resampling with replacement, was used to adjust for non-normality. Interpretation of additive multiple moderation using the PROCESS macro was guided by simple slope analyses performed by PROCESSS. Here the conditional effects of IPV on child self-regulation at different values of the two moderator variables (low, mid, and high supportive responses; low, mid, and high suppressive responses) were calculated and visualized. In determining the low, medium, and high values of the moderator variables, the “16th, 50th, and 84th percentile” feature offered by PROCESS was selected.

#### Model 1.

Model 1, examining supportive and suppressive reactions to children’s emotional displays in the context of psychological aggression, explained 31% of the variance in children’s self-regulation scores, with 12% explained when the supportive × psychological aggression (*R*^*2*^ change = .04, *F* (1,88) = 4.45, *p* = .04) and suppressive × psychological aggression (*R*^*2*^ change = .04, *F* (1,88) = 5.06, *p* = .03) interaction terms were added to the model (see Table 2). A significant interaction effect was observed between psychological aggression and supportive reactions (*β* = .06, 95% CI [.00, .12], *p* = .04) and between psychological aggression and suppressive reactions (*β* = .08, 95% CI [.01, .16] *p* = .03).

**FIGURE 2:**
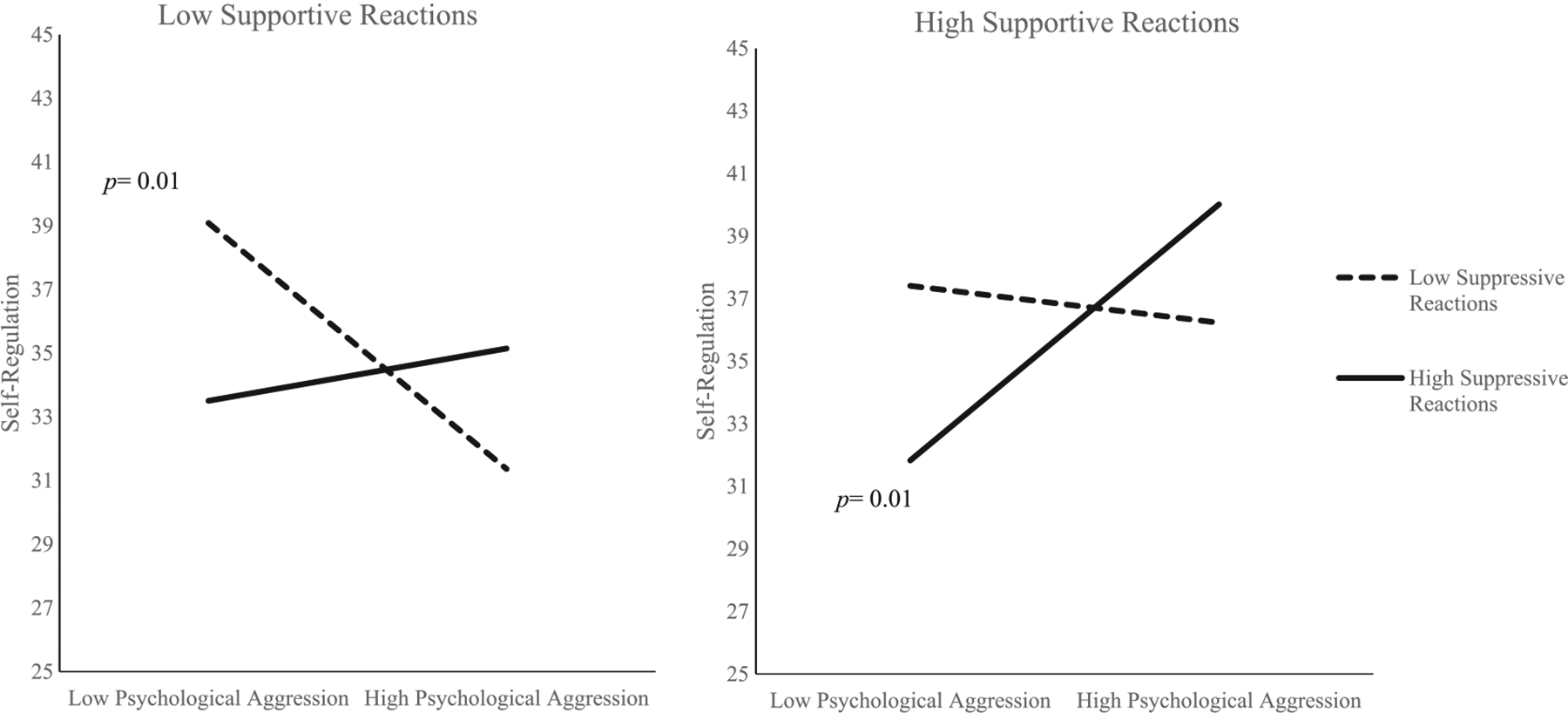
Interaction between psychological aggression and parental reactions in predicting children’s observed self-regulation. *Note*. Values were graphed at low (16th percentile) and high (84th percentile) values for supportive and suppressive reactions. *p* = 0.01.

PROCESS estimated the conditional effect of psychological IPV on child self-regulation for low, medium, and high values of supportive reactions and of suppressive reactions (see Table 3). Analysis of conditional effects demonstrated that the directionality of the relation between psychological aggression and child-self-regulation changed according to level of supportive and suppressive reactions. When mothers reported low levels (<16th percentile) of both supportive and of suppressive reactions, there was a negative relationship between psychological aggression and children’s observer-rated self-regulation (estimate = −.12, 95% CI [−.21, −.03], *p* = .01), such that children exposed to higher frequencies of psychological aggression displayed lower self-regulation. In contrast, when mothers reported high levels (>84th percentile) of both supportive and suppressive reactions, there was a positive relationship between psychological aggression and self-regulation (estimate = .13, 95% CI [.03, .22], *p* = .01), such that children exposed to higher frequencies of psychological aggression displayed higher observer-rated self-regulation (see Figure 2).

#### Model 2.

Model 2, examining suppressive and supportive reactions to emotional displays in the context of physical assault, explained 29% of the variance in children’s self-regulation scores, with 11% explained when the supportive × physical assault (*R*^*2*^ change = .03, *F* (1,88) = 3.44, *p* = .07) and suppressive × physical assault (*R*^*2*^ change = .09, *F* (1,88) = 11.19, *p* < .01) interaction terms were added to the model (see Table 4). The physical assault by supportive reactions interaction term was marginally significant (*β* = .21, 95% CI [−.01, .43], *p* = .07). A significant interaction was observed between physical assault and suppressive reactions (*β* = .35, 95% CI [.14, .55], *p* < .01).

**FIGURE 3:**
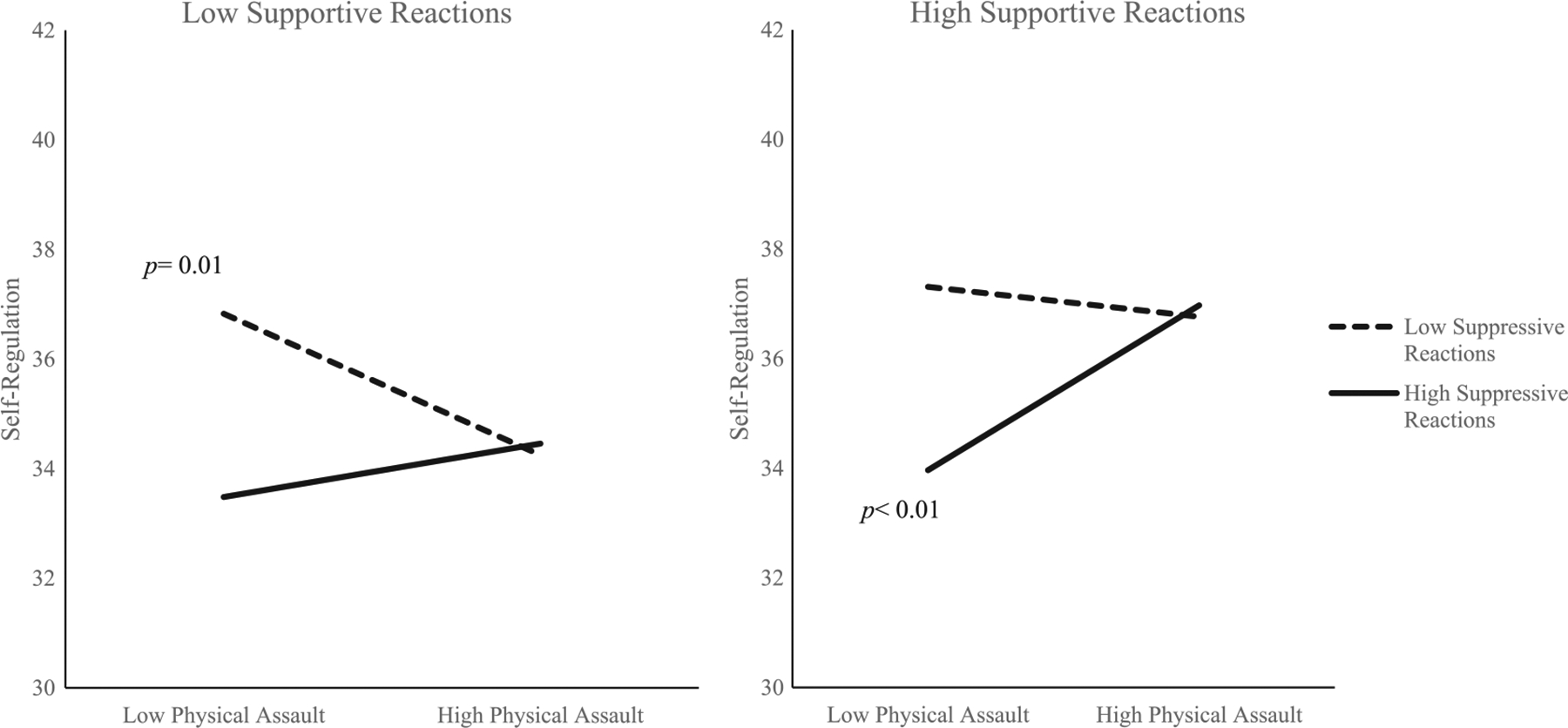
Interaction between physical assault and parental reactions in predicting children’s observed self-regulation. *Note*. Values were graphed at low (16th percentile) and high (84th percentile) values for supportive and suppressive reactions. *p* = 0.01. *p* < 0.01.

Similar to above, PROCESS estimated the conditional effect of physical assault on child self-regulation for low, medium, and high values of supportive reactions and of suppressive reactions (see Table 5). Analysis of conditional effects revealed a shift in the directionality of the relation between physical assault and child self-regulation according to level of supportive and suppressive reactions. When mothers reported low levels (<16th percentile) of both supportive and of suppressive reactions, there was a negative relationship between physical assault and self-regulation (estimate = −.43, 95% CI [−.73, −.13], *p* = .01), such that children exposed to higher frequencies of physical assault displayed lower self-regulation. When mothers reported high levels (>84th percentile) of both supportive and suppressive reactions, there was a positive relationship between physical assault and self-regulation (estimate = .50, 95% CI [.16, .84], *p* < .01), such that children exposed to higher frequencies of physical assault displayed higher self-regulation (see Figure 3).

## 5 | DISCUSSION

Existing literature has identified parental emotion socialization as one of the key mechanisms through which children develop adaptive strategies to regulate their emotions and behaviors (Eisenberg et al., 1998). Additionally, research has found that deficits in self-regulation capabilities in children are associated with various negative physical and mental health outcomes across the lifespan (Eisenberg et al., 2010; Moffit et al., 2011). The goal of the present study was to extend existing literature by examining whether the association between IPV exposure and children’s self-regulation was moderated by parent emotion socialization in a sample of Black mothers.

Our team hypothesized that supportive reactions would have a buffering effect in the context of IPV. This hypothesis was grounded in decades of research establishing that positive parenting can be a source of resilience for children who have witnessed IPV (Fogarty et al., 2019; Graham-Bermann et al., 2009; Howell, 2011; Suzuki et al., 2008). Additionally, we explored the moderating role of suppressive reactions; we did not have an a priori directional hypothesis, given evidence from majority White samples that suppression can be broadly harmful to children’s emotional development (Fabes et al., 2002) but also evidence that suppressive emotion socialization is protective for Black families (Dunbar et al., 2022). Our study adds to a growing body of literature focused on emotion socialization practices in context—including the context of family stress, and the context of Black families, specifically economically marginalized Black families.

In our first model examining psychological aggression, we observed a significant interaction effect between psychological aggression and both supportive and suppressive emotion socialization. In the context of high levels of psychological IPV, children demonstrated higher self-regulation when mothers reported providing supportive emotional reactions, such as validating children’s emotions and engaging in problem-solving behavior. These findings are consistent with research suggesting that parents’ approach to emotion socialization can buffer against the impact of IPV (Katz & Windecker-Nelson, 2006). Specifically, our findings align with extant literature demonstrating that parental awareness and acceptance of children’s emotions are associated with better regulatory outcomes (Eisenberg et al., 1998; Eisenberg, 2020), especially in contexts of family violence (Katz et al., 2016). Although positive associations among supportive parental responses and children’s self-regulation skills are well-documented, associations are predominately explored in White, affluent samples. Our findings suggest that these findings may be generalizable to Black families experiencing poverty, such that supportive parental responses to children’s negative emotions played an adaptive role in children’s self-regulation.

Contrary to past research, we also found that suppressive responses had a buffering effect, changing the directionality of the association between psychological aggression and child self-regulation. For children in households with high levels of suppressive reactions, psychological aggression was positively related to self-regulation, with more IPV exposure associated with higher observed self-regulation. This finding diverges from previous research suggesting that suppression is largely harmful to children’s regulatory development (Morris et al., 2007), but aligns with recent work highlighting the protective role of suppression in Black families (Labella, 2018; Dunbar et al., 2017; Nelson et al., 2013) and the ways that Black parents maybe promote “emotional code switching” as a form of bicultural competence (Lozada et al., 2022). It also supports the broader notion that successful adaptation to traumatic events depends the ability to flexibly enhance or suppress emotional expression in accord with environmental demands (Bonanno et al., 2004; Bonanno & Burton, 2013).

Despite growing interest in emotional suppression in children, the field lacks a guiding framework for understanding how suppressive strategies shape social and emotional functioning and how these processes may differ according to context. In a recent review paper, Gross and Cassidy (2019) speculate that children raised in violent environments may learn that expressing vulnerability is unsafe and become skilled at suppressing future emotional displays. This claim has a strong neurobiological basis: children exposed to early adversity demonstrate accelerated connectivity and maturation in areas of the brain responsible for emotion regulation (Gee et al., 2013). Suppressive reactions therefore may have adaptive value in stressful environments, and children may develop superior regulatory capacities to meet the demands of those environments. Our findings here are consistent with growing evidence that suppressive strategies are adaptive in high-stress environments. Parents may react to children’s emotional expressions in ways that prepare children to manage the demands of those environments.

In the models examining physical assault, we observed similar patterns, with several key differences. Unlike in the models for psychological aggression, there were no significant main effects nor any significant interaction effects of supportive reactions. Results here are not consistent with the original hypothesis, which predicted that supportive reactions would serve as a buffer for both psychological *and* physical IPV. However, it should also be noted that changes in parental emotion socialization may be less influential among children experiencing more extreme, highly visible forms of IPV. For instance, Ehrensaft et al. (2017) found that in the context of more frequent and chronic physical IPV exposure, positive parenting did not affect children’s trauma symptoms, emotional security, or self-regulation. It is possible that witnessing physical IPV is so distressing for young children that any amount of emotional support may fail to improve self-regulatory outcomes. We did, however, continue to see the buffering effects of parents’ suppressive emotion socialization responses on children’s self-regulation in the context of physical IPV. For the reasons discussed above, suppression may offer adaptive advantages in unsafe family environments; when caregiver emotional displays are met with physical harm, for example, suppressive emotional socialization practices may promote more superior self-regulatory capacities (Gross & Cassidy, 2019).

This set of findings also underscores the overlap between self-regulation, behavioral control, and compliance in under-resourced Black families. When Black parents engage in suppressive responses like minimization and punishment, they are setting clear boundaries on children’s emotional expression. Children learn that some expressions are unacceptable to express, and others are not. Compliance to rules and respect for authority holds particular importance to Black parents (Dixon et al., 2008). Black parents are more likely to ascribe to a “no-nonsense” style of parenting (Brody & Flor, 1998) that is characterized by high levels of high levels of control that occur alongside with displays of warmth. Whereas this style of parenting has been misconstrued as “harsh” in some past research, it is a sign of parental involvement and concern in Black families and promotes the development of self-regulation in young Black children (LeCuyer et al., 2011).

Furthermore, research on the effects of parent emotion socialization and children’s self-regulation has largely been limited to White, middle-class samples. As discussed by Eisenberg et al. (1998), emotion socialization may include both “supportive” and “unsupportive” behaviors, but these constructs may be bound within specific cultural contexts; in other words, the categorization of such emotion socialization practices may not be universally applicable to individuals from non-White backgrounds, and researchers should examine parental socialization efforts within specific contexts, attending to both macro- and microsystem influences. Our findings warrant the need for a unified model encompassing both ethnic/racial socialization and emotion socialization together (Dunbar et al., 2017), like in examining emotion socialization processes simultaneously with measures of preparation for bias (Dunbar, Zeytinoglu, et al., 2022). Our findings also add additional, microsystem factors to this ecological conceptualization of emotion socialization, indicating that the family context of violence also influences how parents’ suppressive socialization strategies influence children’s self-regulation.

The current study contributes to the literature in a number of important ways. First, this study highlights the importance of evaluating self-regulation, IPV exposure, and emotion socialization among a sample of low-income, Black families. Results from this study underscore the role of cultural values and race/ethnicity as a key consideration in future studies of emotion socialization. Second, rather than relying on parental report of children’s self-regulation capacities, this study used an ecologically valid, observational measure for self-regulation collected during an assessment task. This approach has a few advantages, namely that it has more relevance to functioning at school, for example (Howard et al., 2019; McCoy, 2019).

Additionally, this study adds to an important new conversation led by Black scholars (Dunbar et al., 2017) about how models of emotion socialization need to be adapted to consider cultural and broader systemic factors, such as structural racism, in considering what patterns of socialization practices are adaptive. Additionally, despite their disproportionate risk for familial and structural violence, Black women are underrepresented in the research literature on IPV (Bradley et al., 2005; Cho, 2012; Mugoya et al., 2020) and even fewer studies seek to articulate familial and culture-specific strengths of Black families that may be protective in the context of family violence.

A key takeaway from this study is that “adaptive” emotion socialization processes are highly context dependent. Given that parents have a profound impact on their children’s emotional well-being and healthy regulatory development, their role is crucial in child mental health interventions. However, importantly, the findings regarding the protective effect of “unsupportive” emotional responses in contexts of high IPV draw critical attention to the cultural relevance of the models of emotion socialization on which family interventions are based. Prevention and intervention work with families affected by familial violence and structural racism needs to pay attention to the cultural and macrosystem factors that contribute to parents’ appraisal of children’s affective expressions.

### 5.1 | Limitations

Despite the study’s contributions to the literature, there are some key limitations. First, the literature is limited by a relatively narrow focus on maternal emotion socialization. In the present study, only the mothers reported on their emotion socialization, which were measured via a self-report questionnaire. The CCNES measures the extent to which parents are reactive to young children’s emotions in a range of distressful situations, but it does not distinguish between public and private settings. It is possible that children might be discouraged to express emotion in public and encouraged to do so at home, or that they might be encouraged to use different strategies with peers versus authority figures (Dunbar et al., 2017). The CCNES also combines responses to a wide range of children’s expressions of negative affect (e.g., sadness, anger, anxiety, and disappointment) into one scale. There is evidence that parents may have different socialization goals and reactions for different emotions (Lougheed et al., 2020; O’neal & Magai, 2005). Further, IPV-exposed mothers may be triggered by or have varying levels of tolerance to different emotional expressions, thereby influencing their response to their child (Loucks & Shaffer, 2014; Stein et al., 2019).

Whereas mothers have been reported to be the primary emotion socializers in children, the inclusion of other primary caregivers (e.g., fathers, grandparents, older siblings) may have provided a unique perspective on how they may impact children’s display of emotions. Thus, future research should consider incorporating other caregiver reports to examine their role in shaping children’s self-regulation capacities, particularly given the importance of kin networks in raising Black children (Dilworth-Anderson, 2019; Hofferth, 1984; Miller-Cribbs & Farber, 2008), and also to examine the impact of concordance or discordance in emotion socialization strategies across multiple caregivers.

Second, the data used in this study are cross-sectional, and thus we are unable to establish causality nor determine the directionality of the processes; longitudinal work is needed in this area, to examine developmental specificity, causality, and the timing of moderating effects. Another limitation was the oversimplification of ethnic identities. Our data did not include a specific examination of whether participants identified with multiple ethnic identities, their history of acculturation, or specific cultural backgrounds. Thus, future research may benefit from investigating proxy variables such as immigration status, spirituality, family structure, and cultural views, which may further elucidate our understanding of differences in emotion socialization behaviors. Finally, our findings and the literature on emotion socialization practices suggest that there are no specific socialization strategies that are always adaptive or maladaptive. Therefore, future research should continue to explore the impact of contextual factors contributing to variability in the nature and function of parental responses to children’s emotions.

Parents play an important role in guiding their children’s emotional development (Eisenberg et al., 1998). The present study examined parental emotional reactions on children’s self-regulatory abilities in the context of IPV, and found a significant association between higher child self-regulation and both “supportive” *and* “suppressive” parental reactions. Our findings are consistent with recent work by Black scholars arguing for a more contextually and culturally situated theory of emotion socialization for Black families (Dunbar et al., 2017), such as Labella (2018) noting the coexistence of Black American families’ beliefs about healthy emotional expression intertwined their historical and ongoing experiences of systematic oppression and racism. Future studies should continue to examine how emotion socialization and racial socialization operate together in family contexts (Dunbar et al., 2017) and examine how these processes’ adaptive implications may vary according to other micro- and macro-systems stressors, children’s stage of development, and other contextual factors.

## Figures and Tables

**TABLE 1:** Descriptive statistics and correlations (Pearson’s *r*) for analytic sample (*n* = 99).

				Aggression type		Reaction type		Self-regulation	Covariates			
	Variable	*M*	*SD*	1	2	3	4	5	6	7	8	9
1.	Psychological aggression	31.60	37.71	–								
2.	Physical assault	5.83	18.51	.53**	–							
3.	Supportive reactions	5.79	1.03	−.12	−.11	–						
4.	Suppressive reactions	2.51	.95	−.02	.00	.13	–					
5.	Child self-regulation	35.04	12.55	−.05	.07	.11	−.01	–				
6.	Child age	51.68	9.20	−.13	.05	.08	.07	.39**	–			
7.	Child sex (0 = male, 1 = female)	50% male	n/a	−.08	−.11	.01	.02	.12	−.02	–		
8.	Maternal education level	3.30	1.55	−.15	−.13	.01	−.11	−.10	−.03	.05	–	
9.	Maternal depression	9.92	10.84	.22*	.07	.01	−.01	−.12	−.13	.10	.11	–

*Note*: *N* = 99.

* *p* < .05 (2-tailed);

** *p* < .01 (2-tailed).

Child sex coded as 0 for male and 1 for female.

**TABLE 2:** Model 1: Psychological aggression and parental reactions predicting child self-regulation.

	*β*	*SE*	*t*	*p*	Lower CI	Upper CI
Constant	31.16	11.62	2.68	.01*	8.07	54.24
Psychological aggression	−.56	.16	−3.51	<.01**	−.88	−.24
Suppressive reactions	−3.27	1.51	−2.16	.03*	−6.28	−.26
Psych × Suppressive	.08	.04	2.25	.03*	.01	.16
Supportive reactions	−1.02	1.38	−.74	.46	−3.77	1.72
Psych × Supportive	.06	.03	2.11	.04*	.00	.12
Child age	.44	.12	3.59	<.01**	.19	.68
Child sex	3.62	2.23	1.62	.11	−.81	8.05
Maternal education level	−1.28	.77	−1.67	.10	−2.81	.24
Maternal depressive symptoms	−.16	.12	−1.34	.18	−.40	.08
Physical assault	.05	.07	.79	.43	−.08	.19

*Note*: *n* = 99.

* *p* < .05;

** *p* < .01.

Child sex coded as 0 for male and 1 for female.

**TABLE 3:** Conditional effects of psychological aggression on child self-regulation at low, mid, and high values of supportive and suppressive reactions.

Suppressive reactions	Supportive reactions	*β*	*SE*	*t*	*p*	Lower CI	Upper CI
Low	Low	−.12	.05	−2.56	.01*	−.21	−.03
Low	Mid	−.05	.05	−.98	.33	−.15	.05
Low	High	−.02	.06	−.30	.76	−.14	.10
Mid	Low	−.06	.04	−1.47	.14	−.14	.02
Mid	Mid	.01	.04	.24	.81	−.07	.09
Mid	High	.04	.05	.90	.37	−.05	.13
High	Low	.03	.06	.44	.66	−.09	.14
High	Mid	.09	.05	2.00	.05*	.00	.19
High	High	.13	.05	2.56	.01*	.03	.22

*Note*: *n* = 99.

* *p* < .05;

** *p* < .01.

Moderator values for supportive and suppressive reactions are estimated at the 16th percentile (low), 50th percentile (mid), and 84th percentile (high).

**TABLE 4:** Model 2: Physical assault and parental reactions predicting child self-regulation.

	*β*	*SE*	*t*	*p*	Lower CI	Upper CI
Constant	17.75	10.63	1.67	.10	−3.37	38.88
Physical assault	−2.02	.70	−2.88	.01*	−3.42	−.63
Suppressive reactions	−1.96	1.22	−1.60	.11	−4.39	.48
Physical × Suppressive	.35	.10	3.35	<.01**	.14	.55
Supportive reactions	.29	1.18	.25	.80	−2.04	2.63
Physical × Supportive	.21	.11	1.86	.07	−.01	.43
Child age	.47	.12	3.79	<.01**	.22	.71
Child sex	4.15	2.27	1.83	.07	−.35	8.66
Maternal education level	−1.05	.77	−1.37	.17	−2.58	.47
Maternal depressive symptoms	−.20	.13	−1.58	.12	−.45	.05
Psychological aggression	.01	.04	.36	.72	−.06	.09

*Note*: *n* = 99.

* *p* < .05;

** *p* < .01.

Child sex coded as 0 for male and 1 for female.

**TABLE 5:** Conditional effects of physical assault on child self-regulation at low, mid, and high values of supportive and suppressive reactions.

Suppressive reactions	Supportive reactions	*β*	*SE*	*t*	*p*	Lower CI	Upper CI
Low	Low	−.43	.15	−2.86	.01*	−.73	−.13
Low	Mid	−.20	.15	−1.31	.19	−.50	.10
Low	High	−.09	.19	−.49	.62	−.46	.28
Mid	Low	−.18	.10	−1.93	.06	−.37	.01
Mid	Mid	.05	.11	.41	.69	−.18	.27
Mid	High	.15	.16	.98	.33	−.16	.47
High	Low	.16	.09	1.76	.08	−.02	.35
High	Mid	.39	.13	3.13	<.01**	.14	.64
High	High	.50	.17	2.90	<.01**	.16	.84

*Note*: *n* = 99.

* *p* < .05;

** *p* < .01.

Moderator values for supportive and suppressive reactions are estimated at the 16th percentile (low), 50th percentile (mid), and 84th percentile (high).

## Data Availability

The data that support the findings of this study are available on request from the corresponding author. The data are not publicly available due to privacy or ethical restrictions.
